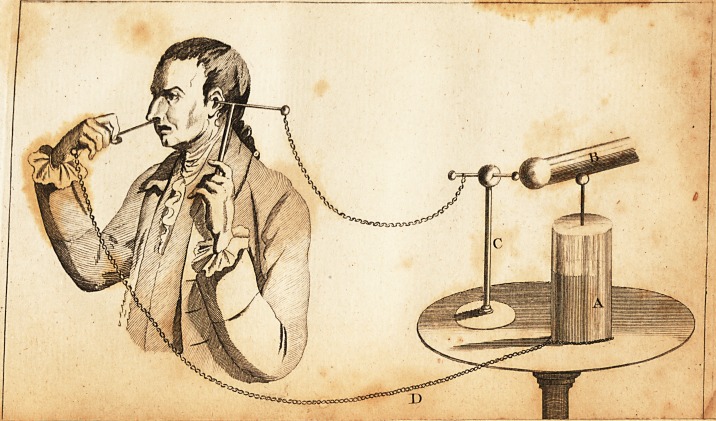# Observations on the Use of Electricity in Deafness

**Published:** 1790

**Authors:** William Blizard

**Affiliations:** Surgeon of the London Hospital


IV. *Obſervations on the Uſe of Electricity in Deafneſs. Communicated in a Letter to Dr.* Simmons *by Mr.* William Blizard, *F. R. S. and S. A. Surgeon of the London Hoſpital.*

TO the organs of ſenſe we are indebted for all our knowledge of the objects that ſurround us. By them we are preſerved from innumerable evils, and rendered capable of various delights. Every attempt, therefore, to preſerve theſe important parts in a ſtate of perfection, or to remedy their defects when impaired, muſt always be conſidered as intereſting to mankind.

The cauſes of deafneſs, and means of relief, have not, I think, been duly conſidered. Perhaps ration performed by Mr. Juſtamond that we alluded. See Vol. IV. of this work, page 282.—EDITOR.

haps the minutenefs and delicacy of the parts conftituting the organ of hearing have difcouraged furgeons from that inveftigation which is neceffary to improvement in the cure of its difeafes; yet whoever fhall induftrioufly examine will difcover as much fimplicity in this as in other organs. We muft ever find a *ne plus ultra*; but do we arrive at it in the ear fooner than in other parts? We are ignorant of the mode of termination of the nerves every where, and know not the precife manner in which impreffions are made on them; but we are convinced that impreffion muft antecede fenfation, and that every thing productive of fenfation is, in refpect to its proper organ, a tangible object.

In hearing, for inftance, the elaftic air, being put into vibratory motion, moves the *membrana tympani*; and by this effect being propagated by the air, in the cavity of the drum, conveyed to thefe parts by the Euftachian tube, correfpondent impreffions, modified probably by the motions of the *officula auditus*, are produced on the expanded *portio mollis* of the feventh pair of nerves, through the medium of a fluid in the feveral parts of the labyrinth.

It

It is manifeſt, then, that, for a perfect condition of the organ of hearing, the *meatus auditorius externus* and *tuba Euſtachiana* muſt be completely open for the paſſage of air, and the nervous expanſion accurately ſuſceptible of impreſſion.

If the latter be not the caſe, there will be a kind of deafneſs, called *nervous*, analogous to that ſpecies of blindneſs termed *gutta ſerena.*

But both in deafneſs and *gutta ſerena*, of this general nature, there are two remarkable diſtinctions, which perhaps have not been duly attended to in practice, and yet their importance, when conſidered a moment, will, I think, be ſtriking. The one is, when the diſeaſe ariſes from an effect anſwering to the idea of contuſion, as when blindneſs happens in an inſtant from a ſudden and too ſtrong influx of light, or as when deafneſs follows a too violent impulſe of the air. The other is, when defect in theſe organs takes place from compreſſive cauſes, as turgeſcency of veſſels, too great a quantity of fluid or matter in their interſtices or parts, ſo as to preſs upon the ſentient nervous extremities: though the effect will be the ſame if the preſſure be in that part of the brain from which the nerves proceed, or upon the nerves before their expanſion. Not long ſince I diſſected an organiſed ſubſtance from the brain of a man who had long been blind, which preſſed upon the origin of the optic nerves.

Manifold experience has taught me, that, in the former deſcription of the caſe, ſtimulants are hurtful. Indeed *à priori* it ſeems plain; for is it reaſonable to ſuppoſe, that if a ſtimulant of the power of 10 has proved too great, and the cauſe of the evil, another of the force of 100 ſhould cure it? In the latter caſe, however, the cauſe of the diſeaſe, if it be from matter out of the circulation preſſing on the nerves, can be removed only through the agency of the abſorbent veſſels; and theſe muſt, therefore, be excited to extraordinary action by ſtimulants. But in what manner are we to ſtimulate parts ſo remote and out of our reach as the internal ear? Electricity has preſented itſelf as a proper agent for this purpoſe, and ſparks have been extracted from the *meatus auditorius externus*; but this has produced little or no effect.

Electricity may be employed, medically, in three ways, viz. firſt, by that kind of ſilent tranſmiſſion transfmiffion of the electric fluid, called *aura electrica*; fecondly, by extracting fparks; and thirdly, by producing fhocks: and thefe methods may be varioufly modified.

The final effect of the firft method has by fome been fuppofed fedative. Experience feems to evince, that, in drawing fparks, the fuperficies only whence the fparks are received is ftimulated; whereas in fhocks the whole extent of the part, between the metallic body which conducts and that which receives the electric fluid, is affected; and, therefore, when we would actuate deeply-feated parts, we ought to employ *fhocks*, properly directed, as fparks can produce but little or no good effect.

Reflections of this kind induced me to think, that in certain cafes of deafnefs, and of obftruction in the Euftachian tube, the electric fhock, being tranfmitted by that tube through the drum of the ear, might prove of utility.

An ingenious ftudent at the London Hofpital, who had for a long time been almoft totally deaf, approved the idea, and determined to have the fuccefs of this method firft tried upon himfelf. I accompanied him to Meffrs. Nairne and Blunt's in Cornhill, where the procefs was obligingly conducted as illuftrated by the annexed plate *.

A. An electrical jar.

B. The conductor.

C. An infulated ftand, or electrometer, from the brafs ball of which a chain proceeds, which is connected to a wire fupported by a ftick of fealing wax in the *meatus auditorius externus*.

D. Another chain placed at the bottom of the jar, joined to a filver wire, which is paffed by the nofe into the Euftachian tube, and fupported by a ftand.

The ball of the electrometer being placed at a greater or lefs diftance from the end of the conductor, will determine the ftrength of the fhock. The fize of the wire for the Euftachian tube fhould be about that of a common filver probe: it fhould be properly curved at the extremity, and made exceedingly fmooth. An eyed probe might ferve very well, or the inftrument commonly ufed for injecting the Euftachian tube. Either of thefe inftruments, being gently introduced by the nofe, will generally pafs readily and without the leaft pain.

* In the year 1784.

B
C
A
D

The experiment was begun with weak ſhocks; but, being repeated daily for ſome time, they were at length employed of a very conſiderable degree of ſtrength. No uneaſineſs or pain in the head was ever produced by this proceſs. It conſtantly occaſioned a tingling ſenſation in the ear, and an immediate amendment in the hearing. This favourable effect would, however, wear away conſiderably in a few hours; yet, by perſevering in the practice, he has at length very nearly acquired his perfect hearing. He had once a ſhock tranſmitted from ear to ear through his head; but the violent pain it occaſioned in the head made him think it unſafe to repeat this experiment.

This caſe of deafneſs having appeared to ariſe from a ſore throat, at a very early period of life, makes it probable that it was the conſequence of obſtruction of the Euſtachian tube.

To aſcertain whether any unpleaſant ſymptom could poſſibly ariſe from tranſmitting the electric ſhock, by the Euſtachian tube, through the ear, I have ſeveral times, in different perſons, had ſhocks given this way, in a more powerful degree than in the above caſe, and have no doubt but that it may always be tried with perfect ſafety: but it certainly ought never to to be employed in an inflammatory or turgid ftate of the veffels of the parts.

This method has been practifed in feveral cafes of deafnefs, both by the above gentleman, and by my own direction, with fuccefs.

It feems a reafonable fuppofition that this mode of procedure might be ufeful in fome cafes of deafnefs arifing from obftruction of the Euftachian tube.

Defective hearing may doubtlefs be the confequence of caufes obftructing the free action of the mufcles fubfervient to, or the mobility of, the *officula auditus:* in which cafes this mode of employing electricity feems particularly calculated to be ufeful.

## Figures and Tables

**Figure f1:**